# Surfactant-Like Behavior for the Adsorption of Mixtures of a Polycation and Two Different Zwitterionic Surfactants at the Water/Vapor Interface

**DOI:** 10.3390/molecules24193442

**Published:** 2019-09-23

**Authors:** Andrew Akanno, Eduardo Guzmán, Laura Fernández-Peña, Francisco Ortega, Ramón G. Rubio

**Affiliations:** 1 Departamento de Química Física, Universidad Complutense de Madrid, Facultad de Ciencias Químicas, Ciudad Universitaria s/n, 28040-Madrid, Spain; drewakanno@gmail.com (A.A.); laura.fernandez.pena@ucm.es (L.F.-P.); fortega@quim.ucm.es (F.O.); 2 Instituto Pluridisciplina, Universidad Complutense de Madrid, Paseo Juan XXIII 1, 28040-Madrid, Spain

**Keywords:** polyelectrolyte, surfactant, adsorption, fluid interfaces, complexation, interfacial properties

## Abstract

The bulk and interfacial properties of solutions formed by a polycation (i.e., poly(diallyl-dimethylammonium chloride), PDADMAC) and two different zwitterionic surfactants (i.e., coco-betaine (CB) and cocoamidopropyl-betaine (CAPB)) have been studied. The bulk aggregation of the polyelectrolyte and the two surfactants was analyzed by turbidity and electrophoretic mobility measurements, and the adsorption of the solutions at the fluid interface was studied by surface tension and interfacial dilational rheology measurements. Evidence of polymer–surfactant complex formation in bulk was only found when the number of surfactant molecules was closer to the number of charged monomers in solutions, which suggests that the electrostatic repulsion associated with the presence of a positively charged group in the surfactant hinders the association between PDADMAC and the zwitterionic surfactant for concentrations in which there are no micelles in solution. This lack of interaction in bulk is reflected in the absence of an influence of the polyelectrolyte in the interfacial properties of the mixtures, with the behavior being controlled by the presence of surfactant. This work has evidenced the significant importance of the different interactions involved in the system for controlling the interaction and complexation mechanisms of in polyelectrolyte–surfactant mixtures.

## 1. Introduction

The physico-chemical behavior of solutions combining charged polyelectrolytes and oppositely charged surfactants both in bulk and close to interfaces—either fluid or solid—has received growing interest in the past two decades [1,2,3,4]. The interaction between polyelectrolyte chains and oppositely charged surfactant molecules in bulk is described as an entropy-driven complexation that leads to the binding of the surfactant molecules to the charged monomers until reaching the isoelectric point, at which compensated complexes present a poor stability in solution. This leads to the separation of the formed complexes from the solution, generally as a solid precipitate. Further increases of the surfactant concentration lead to the formation of overcompensated complexes, and consequently to their resolubilization. This simple mechanism leads to a rich phase behavior in polyelectrolyte–oppositely charged surfactant solutions that can be exploited for the design of different practical applications [5,6,7,8,9,10]. It is worth mentioning that the complexation process is strongly affected by non-equilibrium effects, with the protocol used during polyelectrolyte–surfactant mixing being critical for the properties and structure of the obtained complexes. This is especially important because Marangoni concentration gradients can induce the formation of kinetically-trapped aggregates far from the real equilibrium conformation of the system; furthermore, they present a long-term persistence that determines their significant role in the interaction of polyelectrolyte–oppositely charged surfactant complexes with fluid interfaces [3,11,12,13,14,15,16,17,18,19].

In spite of the extensive research on the behavior of polyelectrolyte–oppositely charge surfactant mixtures, some limitations to their application in the fabrication of consumer products have been introduced by the recent international regulations for the safe and healthy use of chemical compounds [20]. Such regulations recommend the progressive replacement of charged molecules, either polymer or surfactant, for neutral or zwitterionic molecules.

**Figure 1:**
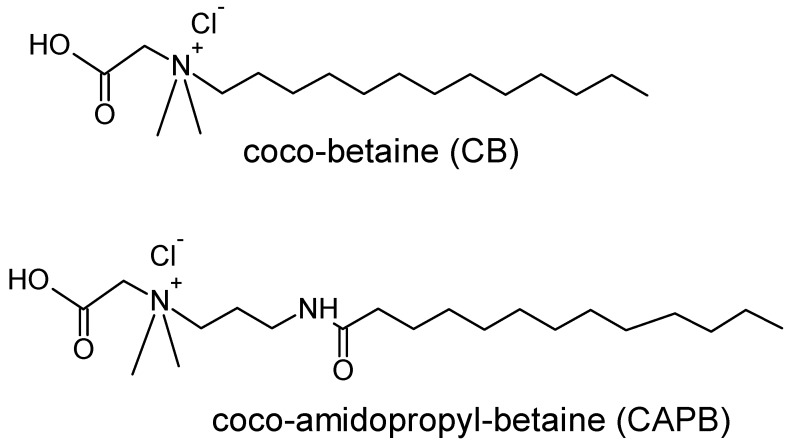
Molecular structures of the two carboxybetaines used in this work.

Therefore, the understanding of the interaction between zwitterionic surfactants and polyelectrolytes is of particular interest because it may be expected that the presence of positively and negatively charged moieties in the surfactant chain may lead to a complexation mechanism similar to that found for polyelectrolyte–oppositely charged surfactant mixtures, enabling the preparation of formulations with better biodegradability and biosafety [21]. However, the complexation mechanism occurring in solutions of polyelectrolytes and zwitterionic surfactants remains less explored than that of solutions containing polyelectrolytes and surfactants bearing opposite charges. Merchán et al. [22] used fluorescence spectroscopy and ζ-potential measurements to probe the interactions in mixture of the zwitterionic surfactant 3-[(3-cholamidopropyl)-dimethyl-ammonium]-1-propanesulfonate (CHAPS) with poly(diallyl-dimethyl-ammonium chloride) (PDADMAC) and poly(sodium styrene sulfonate) (PSS), evidencing the importance of the interactions between the charged monomers and the oppositely charged groups of the surfactant as a driving force of the complexation process, independently of the polyelectrolyte considered. This complexation is suggested to occur by the formation of polyelectrolyte–surfactant micelles either with PDADMAC or PSS. Delgado et al. [23] investigated the interaction of PSS with two different carboxybetaines, combining bulk characterization with surface-sensitive techniques. They found that the formation of complexes in solution occurs as result of the electrostatic interaction between the surfactant and the polymer chains; no effect of such complexes on the interfacial properties of the water/vapor interfaces was reported. This contrasts with the results on the behavior of solutions of polyelectrolytes and oppositely charged surfactants, where a cooperative adsorption of surfactant molecules and polyelectrolyte chains at the water/vapor interface was found by different authors combining different techniques (e.g., surface tension, dilational rheology measurements, neutron reflectometry, ellipsometry) [3,16,17,18,19,24,25,26,27,28]. According to the above discussion, it is clear that the interaction of polyelectrolytes and zwitterionic surfactants deserves further attention. Furthermore, some zwitterionic surfactants, such as the carboxybetaines, are frequently used as foam stabilizers in commercial hair conditioners and shampoos because of their mildness for skin, eyes, and mucosa, which makes them potential candidates for replacing ionic surfactants in consumer products [29,30]. This makes it very interesting to study the behavior of mixtures formed by polyelectrolytes and zwitterionic surfactants. Moreover, the lack of an overall charge of carboxybetaines weakens their interactions with species such as proteins, which makes them suitable for use in biological applications [31]. This work tries to contribute to the knowledge on the physico-chemical bases underlying the behavior of polyelectrolyte–zwitterionic surfactant solutions. For this purpose, we studied the self-assembly in solution and the adsorption to the water/vapor interface of mixtures formed by PDADMAC and two different carboxybetaines. The molecular formulas of the two carboxybetaines, coco-betaine (CB) and coco-amidopropyl-betaine (CAPB), are shown in Figure 1. The two carboxybetaines have the same length of the hydrophobic chain (13 C atoms), differing in size, flexibility, and hydrophilicity of the polar head. It is expected that this study can contribute to the understanding of the most fundamental bases governing the behavior of polyelectrolyte–zwitterionic surfactant solutions. This is important because most of the results found for this type of mixture reflect a contradictory picture. On one side, polyelectrolyte–zwitterionic mixtures evidence the formation of complexes similar to those found for polyelectrolyte–oppositely charged surfactant mixtures. However, on the other hand the aforementioned complexes do not have any interfacial activity [23]. Therefore, it is expected that the combined study of the interaction occurring in solution between a polycation and two chemically different zwitterionic surfactants with the interfacial properties, equilibrium, and dynamics of the layers resulting from the adsorption of the pseudo-binary polymer–surfactant mixtures may help to draw a comprehensive picture of the behavior of this type of system, which presents a growing technological interest.

## 2. Materials and Methods 

### 2.1. Chemicals

PDADMAC with an average molecular weight in the 100–200 kDa range was purchased as a 20 wt% aqueous solution from Sigma-Aldrich (San Luis, CA, USA) and was used as received. The zwitterionic surfactants CB and CAPB were supplied by Clariant International Ltd. (Muttenz, Switzerland) and Solvay S.A. (Brussels, Belgium), respectively. Both carboxybetaines were obtained as aqueous solutions with a surfactant concentration of 31 wt% for CB and 38 wt% for CAPB. CB and CAPB were purified by lyophilization and then recrystallized in anhydrous ethanol (Fisher Scientific, New Hampshire, USA) [32,33]. The purity of the recrystallized product was evaluated using ^1^H NMR.

Ultrapure deionized water used for cleaning and solution preparation was obtained by a multicartridge purification system AquaMAXTM Series. (Young Lin, Gyeonggi-do, South Korea), and had a resistivity higher than 18 MΩ∙cm and a total organic content lower than 6 ppm.

The pH of all solutions was fixed at 5.6 using glacial acetic acid (Sigma-Aldrich, purity > 99%), and the ionic strength was kept constant by adding 40 mM of potassium chloride (KCl) (Sigma-Aldrich, purity > 99.9%). The PDADMAC concentration used for all the solutions was 0.5 wt%, and the surfactant concentration, *c_s_*, was varied in the range from 10^−6^ to 80 mM. The methodology used for solution preparation was adapted from our previous works [25,27,34]. All experiments were carried out at 25.0 ± 0.1 °C.

### 2.2. Techniques

#### 2.2.1. Electrophoretic Mobility

The electrophoretic mobility of the solutions, *μ_e_*, was determined using a Zetasizer Nano ZS from Malvern Instruments Ltd. (Worcestershire, United Kingdom) equipped with a He-Ne laser (*λ* = 632 nm).

#### 2.2.2. Turbidimetry

The turbidity of the solutions was obtained from measurements of their transmittance at 400 nm using a UV–Visible spectrophotometer (HP-UV 8452). Solution turbidity can be defined as *turbidity* = [100 − *T*(%)]/100, where *T* is the transmittance.

#### 2.2.3. Surface Tension Measurements

Surface tension measurements as a function of the surfactant concentration for solutions of the two pure surfactants (CB or CAPB) and their mixtures with PDADMAC were performed using different tensiometers. The adsorption at the water/vapor interface was obtained from dynamic surface tension measurements until a steady state was reached (i.e., changes in surface tension *γ* < 0.1 mN∙m^−1^ for at least 30 min). Special care was taken to limit the evaporation effects. All experimental data is the average of three independent measurements. Further experimental details can be found in our previous publication [25].

1. Surface force tensiometer. A surface force balance from Nima Technology (United Kingdom) fitted with a disposable paper plate-Whatman CHR1 chromatography paper probe was used for measuring the surface tension of the solutions. Additionally, some surface tension measurements were performed using a surface force tensiometer model K10 from Krüss (Germany) with a Pt Du Nöuy ring as contact probe.
2. Drop profile analysis tensiometer. A home-built drop profile analysis tensiometer using the pendant drop configuration was also used for determining the surface tension at the water/vapor interface [35].

#### 2.2.4. Dilational Rheology

A NIMA 702 Langmuir balance from Nima Technology (United Kingdom) was used to measure the surface tension response to sinusoidal changes in the surface area performed following the oscillatory barrier method [36,37]. The dilational viscoelastic moduli $\varepsilon^{*}\;=\;\varepsilon^{\prime }+\varepsilon^{\prime \prime }$ ($\varepsilon^{\prime }$ and $\varepsilon^{\prime \prime }$ represent the dilational elastic and viscous moduli, respectively) were measured in the frequency range of 10^−1^−10^−2^ Hz at an area deformation amplitude Δ*u* = 0.1, which was verified to be an appropriate value to ensure results within the linear regime of the layer response.

## 3. Results and Discussion

### 3.1. Interaction of PDADMAC with Zwitterionic Surfactants in Aqueous Solution

**Figure 2:**
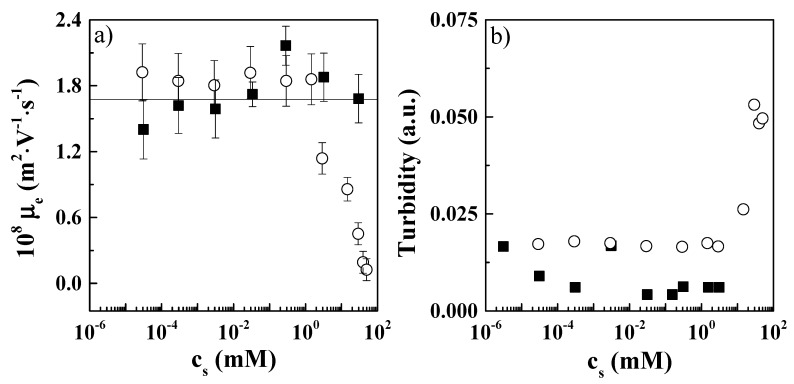
For solutions of poly(diallyl-dimethyl-ammonium chloride) (PDADMAC) and two different zwitterionic surfactants (CB and CAPB): (**a**) surfactant concentration dependence of the electrophoretic mobility (the line shows the electrophoretic mobility of pure surfactant solutions); (**b**) surfactant concentration dependence of the solution turbidity. In both panels: (■) PDADMAC-CB and (○) PDADMAC-CAPB solutions. Results correspond to PDADMAC–surfactant mixtures containing a fixed PDADMAC concentration of 0.5 wt%, left to age for one week prior to measurement.

The interaction between polymers and surfactants in the bulk leads in most cases to the formation of supramolecular complexes. Particularly for systems containing charges, this complexation process is driven by the existence of electrostatic interactions between charged groups of monomers and surfactants, and the van der Waals and hydrogen bond interactions between the different molecules. It could be expected that the presence of anionic and cationic groups in the hydrophilic head of the zwitterionic molecule may in some way affect the aggregation pattern in relation to that found for polyelectrolytes and oppositely charged surfactants [14,15,38,39], even if the binding of surfactant molecules to the polymer chains seem to be guaranteed as a result of the negative charge in the terminal region of the hydrophilic head of the surfactant. The results in Figure 2 obtained from electrophoretic mobility (Figure 2a) and turbidity (Figure 2b) measurements provide the basis for understanding the interaction in solution of PDADMAC and the two zwitterionic surfactants.

**Figure 3:**
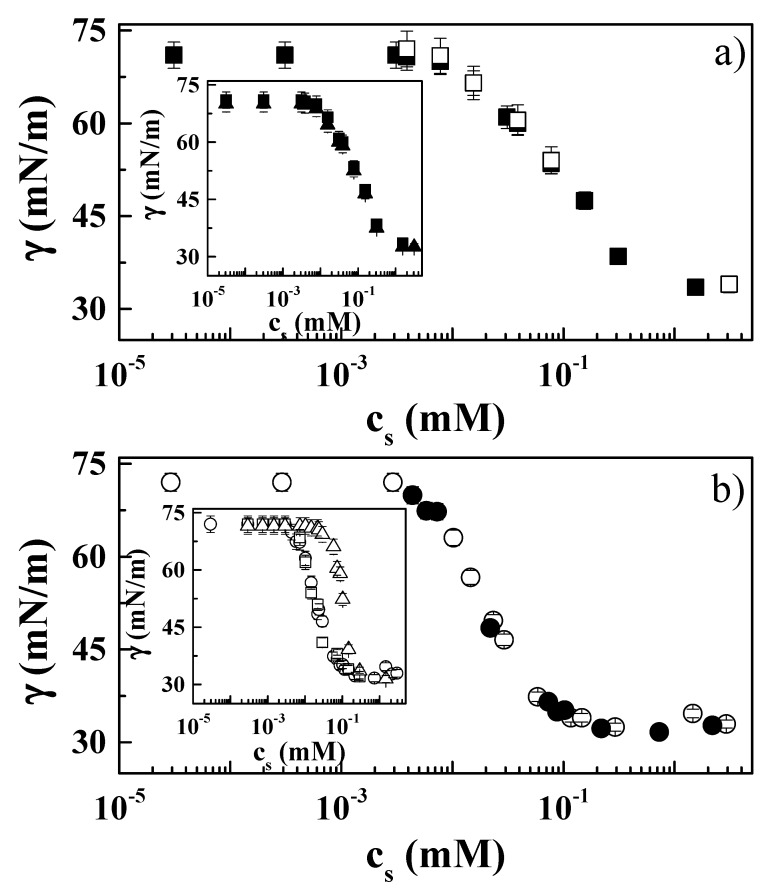
Surfactant concentration dependences of the surface tension measured with a surface force tensiometer fitted with a paper Wilhelmy plate for pure surfactant and PDADMAC–surfactant mixtures: (**a**) CB (□) and PDADMAC–CB (■). (**b**) CAPB (●) and PDADMAC–CAPB (○). Inset panels show comparisons of the surface tension isotherms for PDADMAC–zwitterionic surfactant mixtures obtained using different tensiometers: (■, □) surface force tensiometer fitted with a paper Wilhelmy plate, (●, ○) surface force tensiometer fitted with a Pt Du Nöuy ring, and (▲, ∆) drop profile analysis tensiometer. Results correspond to PDADMAC–surfactant mixtures containing a fixed PDADMAC concentration of 0.5 wt%, left to age for one week prior to measurement.

The results from turbidity and electrophoretic mobility measurements did not show any evidence of complexation between PDADMAC and the zwitterionic surfactants in the low surfactant concentration region, where the values of both electrophoretic mobility and turbidity remained close to those of the polymer solution with the same polymer concentration as the mixtures. This bulk behavior might be interpreted as the existence of a weak interaction, or a lack of interaction, between the species in the solution for the diluted surfactant concentration range. However, the increase of surfactant concentrations in PDADMAC–CAPB solutions provided further insight to deepen the understanding of the complexation mechanism. Figure 2a shows that the electrophoretic mobility dropped from values close to those corresponding to PDADMAC solutions down to values close to the isoelectric point for the highest surfactant concentrations. This was accompanied by an increase of the turbidity (see Figure 2b). These results support the existence of polyelectrolyte–zwitterionic surfactant complexation for the highest surfactant concentrations. This arises from the high concentration of surfactant micelles (critical micelle concentration, cmc, were found to be around 1 mM for CB and 0.3 mM for CAPB using surface tension measurements, see Figure 3) with their negative charge exposed to the solution which can interact with polymer monomers. Thus, surfactant binding occurs through surfactant micelles, enabling an effective compensation of the polymer charges which might be hindered in the low surfactant concentration region due to the role of the positive charge of the surfactant molecules bound to the polyelectrolyte remaining exposed to the aqueous environment.

On the basis of the above results, it is possible to propose two different regimes for surfactant binding in PDADMAC–zwitterionic surfactant solutions: (i) at low surfactant concentrations (high polymer:surfactant (P:S) molar ratio), zwitterionic surfactant molecules bind to the polymer chains without any significant charge neutralization (each neutralized charge is counteracted by the positive charge present in surfactant molecules); and (ii) at high surfactant levels (P:S molar ratio ~ 1), surfactant binding occurs through micelles which enables a real neutralization of PDADMAC charges. Note that experimental evidence for the second regime of complexation is clear. However, important uncertainties exist about the first regime, especially because Pyshkina et al. [40] showed that for mixtures of poly(N-ethyl-4-vinylpyridinium bromide), a polycation, and the zwitterionic surfactant (n-dodecyl-(3-sulfopropyl)ammonium), complexation occurred only when the concentration overcame the cmc of the surfactant. This agrees with the second complexation regime, ruling out the existence of complexation for the lowest surfactant concentrations (*c_s_* < 1 mM).

The absence of complexation signatures in the lowest *c_s_* region does not allow one to rule out the formation of polyelectrolyte–surfactant complexes. This might be easily understood considering the polymer concentration (~0.5 wt%.), which implies that the number of positively charged monomers available was more than 30-fold the number of surfactant molecules available for surfactant concentrations around 1 mM. Hence, assuming the binding of the major part of surfactant molecules to the polymer chain (which was found to be true for anionic surfactants [25,34]), many monomers remain free without interacting with surfactant molecules, thus leading to the formation of undercompensated complexes. Furthermore, the zwitterionic character of CB and CAPB leads to a situation in which the neutralization of the charges of the monomers due to the binding of surfactant molecules is counteracted as result of the presence of a positive charge in the hydrophilic head of the surfactant. Therefore, it may be expected that the binding of zwitterionic surfactants does not modify, at least in an appreciable way, the average charge of PDADMAC chains (see electrophoretic mobility results in Figure 2a), which results in the formation of complexes belonging to an equilibrium one-phase region of the phase diagram. Furthermore, the absence of effective screening of the average charge of the polyelectrolyte chains due to surfactant binding may determine the colloidal stability of the complexes (quasi-null values of turbidity close to pure PDADMAC solutions as shown Figure 2b). These results contrast with those corresponding to mixtures of PDADMAC and different anionic surfactants prepared using similar concentrations and mixing protocol [25,34]. For the latter, even in the one-phase region of the phase diagram, the onset in the two-phase region of the phase diagram was found for surfactant concentrations more than one order of magnitude lower than those corresponding to the equilibrium composition. This phase separation has its origin in the Marangoni stresses arising during the mixing process of polymer and surfactant solutions, which leads to the formation of persistent kinetically trapped aggregates far from the isoelectric point [25,27,34,41,42]. These aggregates remain upon dilution of the samples, yielding a turbidity increase [43]. The absence of this type of aggregate when zwitterionic surfactants are concerned may be because the electrostatic repulsion between the positive charges counteract the aforementioned Marangoni stresses, driving the systems to a complexation mechanism which is reminiscent of real equilibrium.

### 3.2. Adsorption of PDADMAC–Zwitterionic Surfactant Mixtures at the Water/Vapor Interface

The above discussion has evidenced strong differences in the complexation mechanism occurring when zwitterionic surfactants replace anionic surfactants in mixtures with PDADMAC [25,34]. It is expected that such differences could impact the adsorption of the polyelectrolyte–zwitterionic mixture at the water/vapor interface [28,44]. Furthermore, it is expected that surface tension and interfacial dilational rheology measurements will provide further insights on the complexation process existing in PDADMAC–zwitterionic surfactant mixtures. Note that PDADMAC does not present any surface activity for the concentrations used in this study, as shown previously [25,45]. Figure 3 shows the dependences of surface tension on the surfactant concentration obtained for the pure surfactants and their mixtures with PDADMAC.

The surface tension isotherms of both the adsorption of pure surfactant and PDADMAC–zwitterionic surfactant mixtures present a monotonous decay from surface tension values close to the bare water/vapor interface at low surfactant concentrations down to values close to 30 mN/m in the vicinity of the cmc of the pure surfactant. The differences of surface tension for the adsorption of pure surfactant and PDADMAC–surfactant mixtures were smaller than the combined error bars of both mixtures (Figure 3). Together with the previously discussed results for the bulk properties, this seems to confirm the absence of real complexation below the cmc of the surfactant. An explanation of this finding can be given assuming that the presence of the positive charge within the hydrophilic head of the surfactant introduces an electrostatic barrier which may prevent the complexation process between the carboxylic acid group of the surfactants (pK ~ 2–4)—which is expected to be deprotonated under the pH conditions in the present study (slightly acidic, pH ~ 5.5) [46]—and the quaternary ammonium of the PDADMAC monomers.

**Figure 4:**
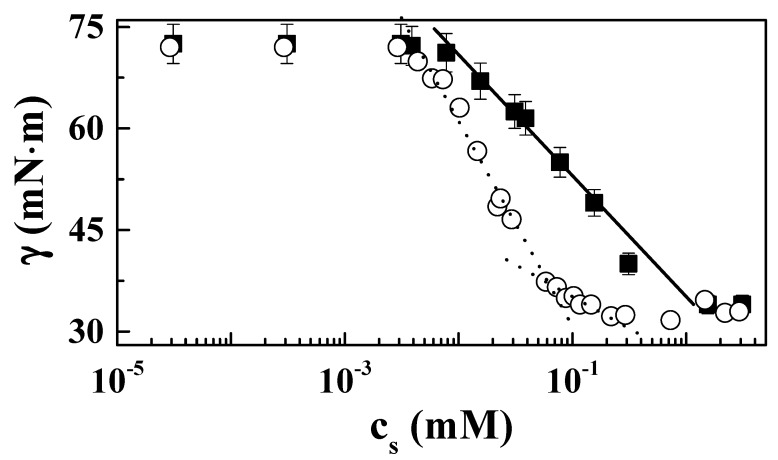
Surfactant concentration dependences of the surface tension measured with a surface force tensiometer fitted with a paper Wilhelmy plate for PDADMAC–surfactant mixtures: PDADMAC–CB (■) and PDADMAC–CAPB (○). The lines represent the different regimes for the surface tension decrease with surfactant concentration. Notice that while only one regime was found for PDADMAC–CB systems (−), two different regimes were found for PDADMAC–CAPB systems (∙∙∙), as evidenced by the change in the slope of the surface tension decay. Results correspond to PDADMAC–surfactant mixtures containing a fixed PDADMAC concentration of 0.5 wt%, left to age for one week prior to measurement.

The analysis of the surface tension isotherms obtained using different tensiometric techniques showed differences when involving different surfactants (see insets in Figure 3). For PDADMAC–CB mixtures (inset Figure 3a), the isotherms obtained with the different techniques used overlapped within the entire surfactant concentration range studied, which indicates the absence of any effect associated with the depletion of material from the solution as a result of the adsorption at the water/vapor interface [25,45]. On the contrary, when mixtures with CAPB (inset in Figure 3b) are considered, the role of the depletion should be taken into consideration, as evidenced by the shifting of the isotherms obtained using a drop profile analysis tensiometer to higher values of surfactant concentration with respect to those obtained using other tensiometers. The differences in the role of the depletion may be related to the different surface activity of CAPB and CB, as pointed out in a recent work that studied the surface tension isotherms of different surface-active materials using several tensiometric techniques [47]. Based on the findings of the mentioned work, a decrease of the surface activity of the surfactant leads to the collapse of the surface tension isotherms obtained using different tensiometers into a master curve [47]. A more detailed analysis of the adsorption behavior of systems containing CB and CAPB indicates additional differences in the behavior of both surfactants. Figure 4 shows a comparison of the surface tension isotherms obtained for mixtures of PDADMAC with the two surfactants.

For CB systems, the surface tension isotherm shows a monotonic decrease of the surface tension with the surfactant concentration. On the other hand, in the case of CAPB, the surface tension isotherm clearly shows two different regimes for the decrease of the surface tension with the surfactant concentration. This is clearer from the different slopes appearing at low and high concentrations in the isotherms, with the slope at the highest surfactant concentration—and probably at the highest surface coverage—possibly associated with the tendency of the CAPB molecule to reorient upon adsorption at the water/vapor interface, which was not observed when CB was the surfactant. This may be rationalized by considering the structure of the CAPB molecule (see Figure 1), with the presence of the amide group in the surfactant molecule enabling the formation of hydrogen bonding with water at the interface. Furthermore, the propyl group between the amide group and the positively charged head group gives some flexibility to the molecule. Therefore, CAPB can form hydrogen bonds with water at the interface at low surface coverage (low surface pressure), occupying larger areas. However, the increase of the surface coverage at high bulk surfactant concentration leads to the reorientation of the absorbed CAPB molecules, leading to a conformation in which molecules align perpendicularly to the water/vapor interface, hence the area occupied by the absorbed CAPB molecule at the interface decreases at higher surface coverage (lower surface tensions).

### 3.3. Interfacial Dilation Rheology of PDADMAC–Zwitterionic Surfactant Mixtures

**Figure 5:**
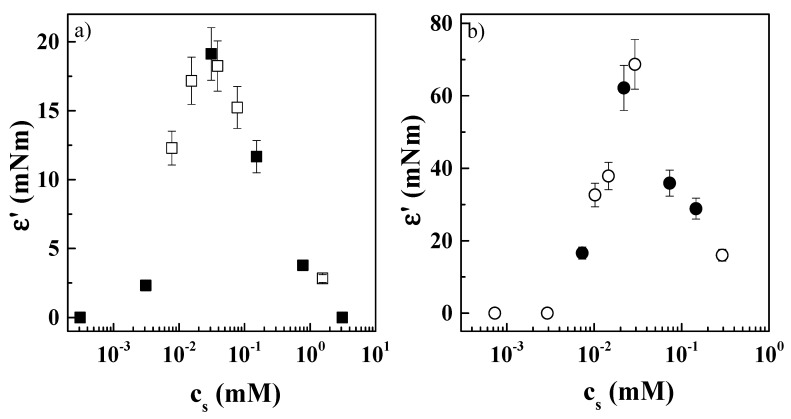
Surfactant concentration dependences of the dilational elastic modulus $\varepsilon^{\prime }$ for pure surfactant and PDADMAC–surfactant mixtures: (**a**) CB (□) and PDADMAC–CB (■). (**b**) CAPB (●) and PDADMAC–CAPB (○). Results correspond to PDADMAC–surfactant mixtures containing a fixed PDADMAC concentration of 0.5 wt%, left to age for one week prior to measurement.

The above discussion was focused on the behavior of the adsorption layers at the fluid interface under steady-state conditions. However, many of the technological applications of this type of system (e.g., the stabilization of thin films, emulsions, or foams) rely on their response to mechanical perturbations [48]. This makes it important to test the response of the surface layers to dilational deformations. In a similar way to what has been found in previous studies of polyelectrolyte–surfactant mixtures [27,34], the elastic modulus, $\varepsilon^{\prime }$, exceeded by several times the viscous one, $\varepsilon^{\prime \prime }$, which in most cases was almost negligible. Therefore, for simplicity, we will only discuss the dependence of the elastic modulus in the following. Figure 5 shows the concentration dependences of the dilational elastic modulus $\varepsilon^{\prime }$ from oscillating barriers experiments at different deformation frequencies.

The results show that for both PDADMAC–zwitterionic surfactant mixtures, the concentration dependences of the dilational surface elastic modulus presented a typical behavior expected for the adsorption of a surface-active molecule at the water/vapor interface [49,50], with $\varepsilon^{\prime }$ growing from values close to zero at the lowest surfactant concentrations (low coverage), up to a maximum, and then decreasing again as the surfactant concentration becomes closer to the surfactant’s cmc. The combined analysis of the results obtained for pure surfactant and PDADMAC–surfactant (Figure 5a,b) mixtures gives further evidence of the absence of an interaction between the polyelectrolyte and the zwitterionic surfactant below the cmc. Indeed, the fact that the dilational surface elastic modulus values were the same for the mixtures and the surfactant means that PDADMAC did not influence the interfacial properties of this mixture. Note that the results obtained here for PDADMAC–zwitterionic surfactant mixtures contrast with those previously obtained for PDADMAC–anionic surfactant mixtures [27,34]. In the latter, the strong binding of surfactant molecules to the polymer chains influenced the behavior of the mixtures at fluid interfaces, leading to a behavior far different from that of the pure surfactant.

### 3.4. Modelling the Interfacial Behavior

**Figure 6:**
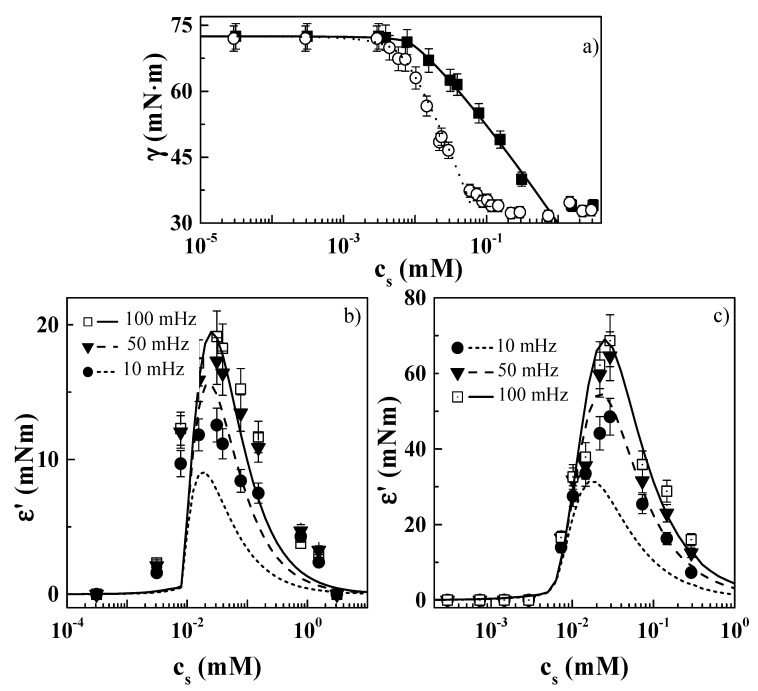
(**a**) Surfactant concentration dependences of the surface tension measured with a surface force tensiometer fitted with a paper Wilhelmy plate and theoretical isotherms (lines) obtained using a reorientation model including the compressibility of the layer for: PDADMAC–CB (■) and PDADMAC–CAPB (○) mixtures. (**b**,**c**) Experimental (symbols) and calculated (lines) concentration dependences of the dilational elastic modulus $\varepsilon^{\prime }$ for PDADMAC–CB and PDADMAC-CAPB, respectively. Results correspond to PDADMAC–surfactant mixtures containing a fixed PDADMAC concentration of 0.5 wt%, left to age for one week prior to measurement.

**Figure 7:**
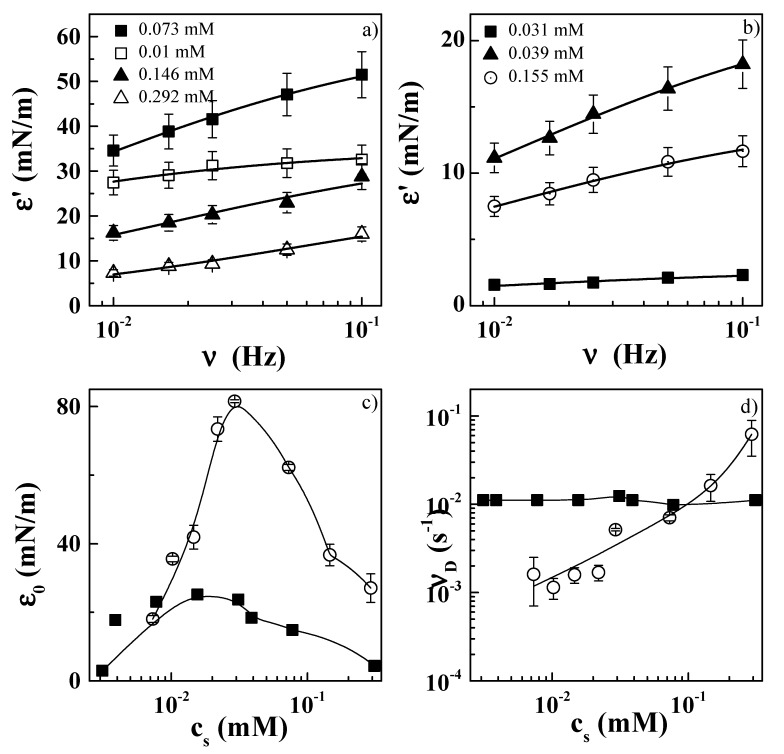
(**a**,**b**) Frequency dependences of the interfacial dilational elastic modulus for PDADMAC–CB and PDADMAC–CAPB mixtures, respectively. Symbols and lines represent experimental results and the best curves obtained in the framework of the Lucassen–van der Tempel equation [56]. (**c**,**d**) Concentration dependences of the limit of elasticity, *ε*_0_, and the characteristic frequency, *ν_D_*, respectively, for PDADMAC–CB (■) and PDADMAC–CAPB (○). Results correspond to PDADMAC–surfactant mixtures containing a fixed PDADMAC concentration of 0.5 wt%, left to age for one week prior to measurement.

The dependences of surface tension (Figure 6a) and elastic modulus $\varepsilon^{\prime }$ (Figure 6b,c) on the surfactant concentration for PDADMAC–zwitterionic surfactant mixtures are well described in terms of a reorientation model accounting for the compressibility of the surface layer (see Figure 7c,d). The model considers that adsorbed molecules can exist at the fluid interface in two different orientations, with the area occupied by molecules at the interface, ω, changing continuously with the surface pressure (Π = *γ* − *γ*_0_, with *γ* and *γ*_0_ being the surface tensions of a given solution and the bare water/vapor interface, respectively) according to [51,52,53,54]:

(1) $$\omega \;=\;\omega_{0}(1\;-\;\varepsilon_{c}\Pi \theta ),$$

with $\omega_{0}$ being the area occupied by a solvent molecule, θ = ΓΠ, with Γ representing the surface excess (i.e., the interfacial coverage), and $\varepsilon_{c}$ the relative two-dimensional compression ratio of the surfactant molecules in a packed interfacial film. The reorientation model considers that molecules can be adsorbed at the fluid interfaces with different tilting angles, generally in two different states (with the orientation at low coverage being referred to as state 1, and the conformation at high coverage defined as state 2), assuming a perpendicular orientation at high values of interfacial coverage [54]. This can be described by the following equation of state:

(2) $$\Pi =-\frac{RT}{\omega }\ln\left(1-\theta \right),$$

and the adsorption isotherm

(3) $$\Pi =\frac{\Gamma_{2}\omega }{{\left(1-\theta \right)}^{\omega_{2}/\omega }},$$

with *b* being the adsorption equilibrium constant and $\Gamma_{2}$ and $\omega_{2}$ the surface excess and the area of molecules in state 2, respectively. The total surface excess can be defined as:

(4) $$\Gamma =\Gamma_{1}+\Gamma_{2},$$

(5) $$\omega \Gamma =\theta =\omega_{1}\Gamma_{1}+\omega_{2}\Gamma_{2},$$

with $\Gamma_{1}$ and $\omega_{1}$ the surface excess and the area of molecules in state 2. Note that, as expected for a diluted film, $\omega_{1}$ > $\omega_{2}$. A last aspect to define is the ratio between the surface excess of molecules corresponding to the two different states:

(6) $$\frac{\Gamma_{1}}{\Gamma_{2}}=exp\left(\frac{\omega_{1}-\omega_{2}}{\omega_{2}}\right){\left(\frac{\omega_{1}}{\omega_{2}}\right)}^{\alpha }exp\left(-\frac{\Pi \left(\omega_{1}-\omega_{2}\right)}{RT}\right).$$

with *α* being a constant accounting for the conditions in which the number of molecules in state 1 could exceed that in state 2. The use of a reorientation model considering the contribution of the compressibility of the interfacial layer provides a good description of the behavior of both PDADMAC–CB and PDADMAC–CAPB mixtures at the water/vapor interface. It is possible to use a theoretical model developed for the description of the behavior of surfactants because of the negligible contribution of the PDADMAC to the interfacial behavior. The results obtained in this work agree with those obtained by Fainerman et al. [55], who reported the possible adsorption of carboxybetaines at fluid interface in more than one conformation.

Table 1 summarizes the parameters obtained from the analysis of the experimental data using the aforementioned model. The results show that the area occupied by each adsorbed molecule at low surface coverage, *ω*_1_, was higher for PDADMAC–CAPB systems, in agreement with the suggestion made in Section 3.2. This can be rationalized according to the results obtained by Fainerman et al. [55], considering that at low surface coverage the more asymmetric carboxybetaine molecules occupied larger areas at the fluid interface. The similar value found for $\omega_{2}$ evidences that at the highest values of the coverage the hydrocarbon tails of both CB and CAPB were tilted to the same degree upon adsorption at the interface. Furthermore, the interaction parameter *a* and the adsorption equilibrium constant *b* were higher for the PDADMAC–CAPB system than for the PDADMAC–CB system, confirming the high surface activity of the CAPB molecules at the water/vapor interface as seen from the higher values of the dilational elastic modulus.

The negligible interaction between PDADMAC and zwitterionic surfactants leads to important differences in the relaxation mechanism involved in the equilibration of the interfacial layer compared to the behavior reported for mixtures of PDADMAC and anionic surfactants. Here, the relaxation mechanism can be described in terms of the Lucassen–van den Tempel model [56] accounting for the diffusion of the surfactant molecules at the interface, whereas for PDADMAC–anionic surfactant mixtures additional relaxation mechanisms were necessary for a good description of their behavior [27,34,57,58]. Thus, the relaxation mechanism of PDADMAC–zwitterionic surfactant layers can be described by the following expression:

(7) $$\varepsilon^{\prime }=\varepsilon_{0}\frac{1+\zeta }{1+2\zeta +2\zeta^{2}},$$

where ζ=νDν, with *ν* and *ν_D_* being the deformation frequency and the characteristic frequency of diffusion, respectively, and *ε*_0_ representing the limit value of the elasticity, corresponding to a monolayer of a soluble surfactant to the Gibbs limit. Figure 7 shows the frequency dependences of the interfacial dilational elastic modulus for PDADMAC–CB (Figure 7a) and PDADMAC–CAPB (Figure 7b) mixtures with different surfactant concentrations.

The parameters obtained from the analysis of the data in the framework of the Lucassen–van den Tempel model for both mixtures are shown in Figure 7c,d [56]. The limit surface elasticities *ε*_0_ showed similar trends for both mixtures, with higher values for PDADMAC–CAPB mixtures, in agreement with the higher surface activity of CB. The characteristic frequency, *ν_D_*, associated with the relaxation process of the adsorbed surface layers of both mixtures appeared to be within the range 0.01–100 s^−1^. This frequency range is similar to that reported for the diffusion of low-molecular-weight surfactants at the air/liquid interface [50]. The concentration dependences for the characteristic diffusion frequency *ν_D_* were strongly affected by the considered surfactant. For the PDADMAC–CB system, *ν_D_* appeared to be rather independent of the surfactant concentration, whereas for the PDADMAC–CAPB system an increase was observed at the highest surfactant concentrations. This may explained considering $\nu_{D}=\frac{D}{2\pi }{\left(\frac{dc}{d\Gamma }\right)}^{2}$, and the shapes of the adsorption isotherms (Figure 4), with the increase of *ν_D_* for the highest surfactant concentration likely being related to the change in the orientation of the surfactant molecules at the interface, evidenced by the change in the slope (*dγ*/*dc*) of the adsorption isotherm. Thus, the absence of this increase in PDADMAC–CB is easily justified, assuming the constant slope in the adsorption isotherm. The existence of a reorganization of the molecules of the surfactant for the highest surfactant concentration in PDADMAC–CAPB systems (see surface tension isotherm in Figure 4) should be associated with the appearance of a more complex relaxation spectrum than that described by the Lucassen–van der Tempel model. However, the limited frequency range accessible made it impossible to probe the surfactant reorganization at the interface using the oscillatory barrier method.

## 4. Conclusions

This work was focused on the elucidation of the behavior of mixtures formed by PDADMAC and two different zwitterionic surfactants (CB and CAPB) in bulk and upon their adsorption at the water/vapor interface. Turbidity and electrophoretic mobility measurements suggested a lack of aggregation in solutions formed by PDADMAC and the zwitterionic surfactants for concentrations below the cmcs of the surfactants. The absence of surfactant binding to the polyelectrolyte chains was confirmed with studies on the adsorption of polyelectrolyte–zwitterionic surfactant mixtures at the fluid interface, with the interfacial properties of these mixtures being similar to those of pure surfactant. Based on the obtained results, it was possible to rule out the interaction of PDADMAC and zwitterionic surfactant in solution, which means that the interfacial behavior was dominated only by the specific nature of the surfactant, with the polyelectrolyte playing a negligible role in the interfacial behavior. The results indicate important differences from the behavior previously reported for PDADMAC–anionic surfactant mixtures, where a strong binding of the surfactant molecules to the polyelectrolyte chains was found, significantly affecting the interfacial behavior of such mixtures [42]. Therefore, the obtained results for PDADMAC–zwitterionic mixtures were unexpected because the presence of negatively charged groups located in the terminal region of the hydrophilic charged heads of the zwitterionic surfactants make possible the electrostatic binding of the surfactant molecules to the polyelectrolyte chains. Thus, it may be concluded that the influence of the electrostatic repulsion associated with the ammonium group in the surfactant molecules can hinder the association between PDADMAC and zwitterionic surfactants.

## Figures and Tables

**Table 1:** Parameters obtained from the fitting of the experimental results to the reorientation model considering the role of the interfacial compressibility.

	PDADMAC–CB	PDADMAC–CAPB
10^−5^*ω*_1_ (m^2^/mol)	1.2	0.25
10^−5^*ω*_2_ (m^2^/mol)	2	2
*a*	7	5
*α*	1.2	0.9
10^3^*ε* (m/mN)	3.70	0.02
*b* (L/mmol)	0.56	3.24 × 10^−5^
